# Diagnostic and Therapeutic Applications of Exosomes in Lung Cancer

**DOI:** 10.3390/cells15070632

**Published:** 2026-03-31

**Authors:** Disha Nagesh Moholkar, Raghuram Kandimalla, Margaret Wallen, Kavitha Yaddanapudi, Ramesh Gupta, Farrukh Aqil

**Affiliations:** 1 Brown Cancer Center, University of Louisville, Louisville, KY 40202, USAraghuram.kandimalla@louisville.edu (R.K.); kavitha.yaddanapudi@louisville.edu (K.Y.);; 2 Department of Pharmacology & Toxicology, University of Louisville, Louisville, KY 40202, USA; 3 School of Natural Sciences, Department of Biology, Indiana University Southeast, New Albany, IN 47150, USA; wallenm@iu.edu; 4 Department of Microbiology and Immunology, University of Louisville, Louisville, KY 40202, USA; 5 Department of Surgery, University of Louisville, Louisville, KY 40202, USA; 6 3P Biotechnologies, Inc., Louisville, KY 40202, USA; 7 Department of Medicine, University of Louisville, Louisville, KY 40202, USA

**Keywords:** exosomes, lung cancer, clinical applications, diagnostics, targeted therapy

## Abstract

Lung cancer remains one of the leading causes of cancer-related mortality worldwide, with a five-year survival rate of only 26%, primarily due to late-stage diagnosis and limited treatment options. Exosomes, nanosized extracellular vesicles released by nearly all cell types, have emerged as promising tools in both diagnostics and therapeutics. Their unique composition containing proteins, lipids, and nucleic acids reflects the molecular profile of their cell of origin, making them excellent candidates for non-invasive early detection biomarkers. For therapeutic applications, exosomes offer biocompatible, low-immunogenicity platforms capable of delivering diverse therapeutic agents, including small molecules, siRNAs, and antimetabolites, directly to tumor cells while minimizing systemic toxicity. Functionalization strategies, such as folic acid tagging, have further enhanced tumor specificity, especially in cancers with high folate receptors. However, clinical translation is hindered by challenges including lack of standardized isolation and characterization methods, high production costs, and regulatory uncertainties. Despite these limitations, ongoing research continues to optimize exosome production, targeting, and integration with conventional therapies. Milk- and colostrum-derived exosomes have shown promising potential due to their abundance, scalability, oral bioavailability, and safety. Collectively, exosomes represent a transformative approach in lung cancer management, with the potential to improve early diagnosis, enhance therapeutic efficacy, and reduce adverse effects, thereby offering a path toward more personalized and effective cancer care.

## 1. Introduction

Cancer has become a critical global health issue, with alarming mortality rates. Lung cancer is among the most aggressive and prevalent subtypes, affecting both sexes worldwide. Despite advances in treatment strategies, the incidence and mortality of lung cancer have continued to rise, with an estimated 229,410 new cases and 124,990 deaths projected for 2026 in the United States alone [1]. Notably, in 2021, lung cancer incidence in women under 65 surpassed that in men (15.7 vs. 15.4 per 100,000; RR, 0.98; *p* = 0.03) [2]. Lung cancer accounts for approximately 25% of all cancer-related deaths, with more than 80% directly attributed to cigarette smoking [3]. Major risk factors include tobacco use, environmental exposures (such as biomass fuel, arsenic, radon, industrial carcinogens, and air pollution), and oncogenic driver mutations [2]. Between 2011 and 2016, smoking (both current and former) was associated with 90% of lung cancer cases in men and 84% in women [4]. Histologically, lung cancer is classified into non-small-cell lung carcinoma (NSCLC), small-cell lung carcinoma (SCLC), mesothelioma, sarcoma, and carcinoid tumors. NSCLC and SCLC constitute approximately 90% of all lung cancer cases, while other forms are relatively rare [5]. NSCLC is further subdivided into adenocarcinoma, squamous cell carcinoma, and large cell carcinoma, each characterized by distinct histopathological features, molecular alterations, and clinical outcomes.

Over the past decade, the therapeutic landscape of NSCLC has evolved considerably with the development of targeted therapies directed against key oncogenic drivers such as EGFR mutations and ALK and ROS1 rearrangements, along with recently targetable KRAS mutations, which have significantly improved clinical outcomes in molecularly defined subgroups of patients. In parallel, immunotherapy has emerged as a transformative approach, particularly through immune checkpoint inhibitors targeting PD-1/PD-L1 and CTLA-4 pathways, resulting in improved survival responses. Furthermore, combination strategies integrating chemotherapy, targeted therapy, and immunotherapy are increasingly being explored to overcome resistance and enhance therapeutic efficacy. Despite these advances, challenges such as tumor heterogeneity, acquired resistance, and variability in patient response continue to limit long-term success, highlighting the need for innovative and more effective therapeutic approaches [6,7,8].

Conventional treatments including chemotherapy, radiotherapy, and surgical resection, either alone or in combination, remain the cornerstone of lung cancer management [9]. However, chemotherapeutic agents often lack selectivity, damaging healthy tissues, while radiotherapy may also hamper surrounding normal cells. Treatment regimens typically involve cyclic drug administration with interval periods to allow recovery of normal tissues. Unfortunately, these intervals in chemotherapy regimens can also enable cancer cells to recover, potentially resulting in drug resistance, recurrence, or myelosuppression [5]. To address these challenges, researchers have developed a variety of drug delivery systems (DDS), including nanoparticles, dendrimers, carbon dots, and lipid-based carriers [10]. Among these, lipid-based DDS are widely studied due to their ability to provide controlled and targeted drug release, enhance bioavailability, and solubility, protect payloads from degradation, and exhibit high biocompatibility [11]. One of the most promising lipid-based DDS are the exosome or small extracellular vesicles (sEVs).

Extracellular vesicles (EVs) represent a heterogeneous population of membrane-bound vesicles released by cells, broadly classified into exosomes, microvesicles, and apoptotic bodies based on their size and biogenesis. Exosomes, also referred to as small extracellular vesicles (sEVs), typically range from 30 to 150 nm in diameter and originate from the endosomal pathway. In this review, the term “exosomes” is used specifically to denote these small EVs unless otherwise indicated, in accordance with current International Society for Extracellular Vesicles (ISEV) guidelines.

**Figure 1:**
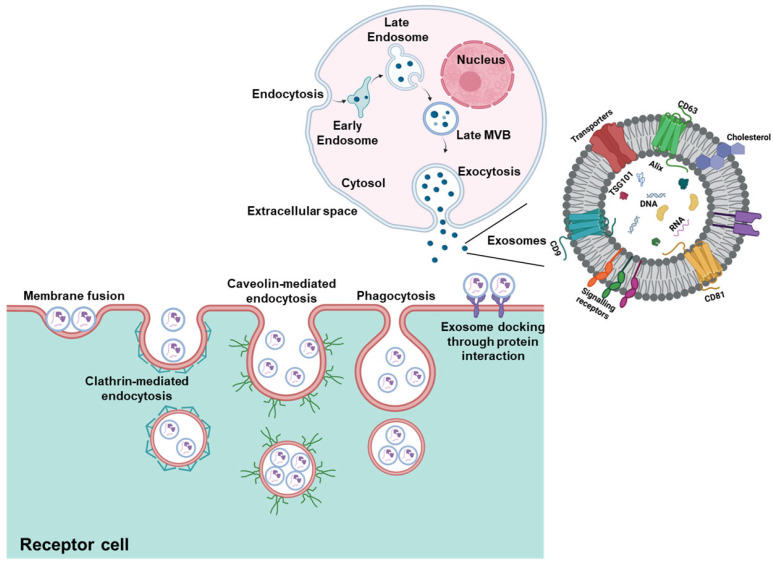
**Exosome Biogenesis, Structure, and Cellular Uptake Mechanisms.** Overview of exosome biogenesis, molecular architecture, and modes of cellular uptake. The top panel illustrates exosome formation within multivesicular bodies (MVBs), where intraluminal vesicles bud into the endosomal lumen. Upon fusion of MVBs with the plasma membrane, exosomes are released into the extracellular space. The right panel depicts the exosomal lipid bilayer, highlighting surface markers (tetraspanins: CD9, CD63, CD81; integrins; MHC), internal cargo (proteins, nucleic acids, enzymes, and lipids), and associated functional proteins (TSG101, ALIX). The bottom panel shows multiple pathways of exosome uptake by recipient cells, including membrane fusion, clathrin-mediated endocytosis, caveolin-mediated endocytosis, phagocytosis, and protein-mediated docking and internalization.

Exosomes are naturally secreted, nanoscale vesicles containing a complex cargo of biomolecules (proteins, miRNA, DNA, etc.). According to the ExoCarta database (http://www.exocarta.org (accessed on 7 February 2025)), exosomes carry 13,472 proteins, 3408 mRNAs, 10,755 miRNAs, and 3946 lipids [12]. Their composition is influenced by the cell of origin and the surrounding microenvironment [13]. Exosomes originate from the endocytic pathway, beginning with the formation of early endosomes through membrane invagination (Figure 1). Intraluminal vesicles (ILVs) form within multivesicular bodies (MVBs), which then mature and fuse with the plasma membrane to release ILVs as exosomes into the extracellular space [14]. Exosomes play vital roles in biological processes including cell-to-cell communication [13]. They interact with recipient cells through membrane adhesion, fusion, receptor-mediated endocytosis, or phagocytosis [15]. Unlike traditional signaling modes (paracrine, endocrine, exocrine, or synaptic) that rely on passive diffusion, exosomes enable precise and widespread message delivery in complex scenarios [16]. This function is facilitated by characteristic proteins such as Rab, GTPases, and tetraspanins (CD9, CD63, CD81, CD82) [17].

Physiologically, exosomes contribute to immune responses, cell migration, tissue repair, angiogenesis, cellular differentiation, homeostasis, and waste removal [13,18,19]. A key function is their ability to transfer bioactive molecules from donor to recipient cells, thereby altering recipient cell behavior [20]. In pathological contexts, exosomes can serve as biomarkers that reflect the state of originating cells and offer insights into disease development and progression, including cancer [13,21].

Exosomes are secreted by nearly all cell types and are found in biological fluids such as blood/plasma, milk, urine, saliva, cerebrospinal fluid, synovial fluid, amniotic fluid, bronchoalveolar lavage, pleural effusion, ascites, and semen [22]. Because the molecular content of exosomes reflects the originating cell type, they hold promise as highly specific diagnostic tools [23]. In cancer, circulating tumor-derived exosomes can carry oncogenic signatures useful for diagnosis, monitoring disease progression, and evaluating therapeutic response [23]. Similarly, CNS-derived exosomes that contain amyloidogenic proteins have been explored for early diagnosis of neurodegenerative disorders such as Alzheimer’s disease, Parkinson’s disease, and ALS [24,25]. As of late 2022, 59 clinical trials had been registered for exosome-based therapeutics. The most commonly targeted diseases include lung conditions (11 trials), SARS-CoV-2 (9), and cancer, cardiovascular, and neurological disorders (4 each) [26]. Additionally, exosomes are being explored for diagnostic applications in over 200 clinical trials, with approximately 108 focused on cancer detection, followed by neurological (15), cardiovascular (13), and lung diseases (6) [26].

Existing literature on the potential of exosomes in lung cancer scenarios focuses on tumor-derived exosomes as mediators of oncogenic signaling, therapy resistance and liquid-biopsy biomarkers for either diagnostic, prognostic, or therapeutic applications [27]. Alternatively, this review discusses the potential of exosomes from different origins to be used as drug delivery vehicles [28]. This review uniquely focuses on exosomes in the context of lung cancer by integrating their diagnostic, prognostic, therapeutic, and immunotherapeutic roles within a single, coherent framework, thereby capturing the full diagnostic to therapeutic continuum in a lung cancer–oriented context. It further adopts a translational lens by systematically connecting mechanistic and preclinical findings with ongoing and completed clinical trials, thereby emphasizing how exosome-based strategies are progressing toward real-world implementation in lung cancer management. Uniquely, this article incorporates a substantial body of work generated by our own laboratory, which has pioneered and developed milk- and colostrum-derived exosome-based technology as scalable, orally deliverable, and clinically viable delivery systems, an emerging but underrepresented area that is rarely addressed in depth in current reviews, positioning this article as a distinct and forward-looking contribution to the exosome literature. Building on the pioneering work from our own laboratory in developing milk exosome-technology, much of which is critically discussed here, this review aims to provide a unique, forward-looking perspective that serves as a translational roadmap for future exosome-based interventions in lung cancer.

## 2. Methods of Exosomal Isolation

Exosome isolation remains a critical determinant of downstream applications; however, it is a well-established area with extensive methodological coverage in literature. Therefore, this section provides a concise overview of the most commonly used isolation techniques, with emphasis on their translational relevance, scalability, and impact on exosome purity and functionality, rather than detailed procedural descriptions.

A recent article provides a comprehensive head-to-head comparison of nine plasma exosomes isolation methods, evaluating their yield based on purity and physicochemical parameters such as size distribution and proteomic profiles to help select the best method for their downstream applications [29]. Ultracentrifugation is still widely regarded as the “gold standard” for exosome isolation in many recent studies applying differential and density-gradient protocols to handle large volume samples even though issues such as shear-related vesicle damage and co-purified protein aggregates remain a concern [29,30]. Density gradient centrifugation utilizes sucrose- or iodixanol-based gradients of varying densities to isolate exosomes with high purity [31].

Size-exclusion chromatography (SEC) resolves EVs based on hydrodynamic size and is minimally disruptive, preserves exosome integrity, making it suitable for downstream applications [29]. Researchers isolated exosomes from patient plasma suitable for SERS analysis. The assay accurately identified the tissue of origin for different early-stage cancers, including lung cancer [32]. Ultrafiltration is a membrane-based technique that separates exosomes using molecular weight cut-off filters with pore sizes ranging from 10 to 300 kDa. This method is simple and does not require specialized equipment, although it can be time-consuming. It is often used as a pre-concentration or intermediate step before SEC, or as an additional step to reduce contamination [29,31]. Tangential flow filtration (TFF) has emerged as an efficient and scalable method for exosome isolation [33]. The tangential flow across the membrane surface minimizes clogging as observed in ultrafiltration and enhances filtration efficiency, making it a promising method for diagnostic use [34].

Magnetic affinity-based isolation techniques such as MagNet (strong anion exchange) and MagCap (Tim4-PS affinity) are increasingly being used for exosomal isolation [29]. These techniques are part of industrial processes used for isolation of biomarkers from complex samples. Thus, exosome assays can be readily transferred to existing platforms once the clinical utility of exosomes is confirmed [35]. Alternatively, crowding agents such as polyethylene glycol (PEG), dextran, protamine, acetate and high-ionic-strength salts (e.g., ammonium sulfate) are used to alter EV solubility, forming aggregates that can be pelleted by low-speed centrifugation [29,36,37].

In summary, high-resolution isolation techniques such as density gradients, size-exclusion chromatography, and magnetic affinity-based approaches effectively eliminate lipoprotein particles and soluble plasma proteins. These techniques enhance the analytical outcomes and improve inter-study reproducibility of downstream analysis such as proteomics and transcriptomics analysis. Conversely, chemical precipitation and ultracentrifugation are high-throughput but lower-selectivity methodologies that often co-isolate non-vesicular protein aggregates and lipoprotein complexes. This can lead to inter-laboratory variability and compromise biomarker validation. For clinical translatability, the implementation of scalable yet non-denaturing purification steps such as tangential flow filtration and ultrafiltration is critical to maintain structural and functional integrity of isolated exosomes. These approaches are essential for manufacturing consistency and adherence to GMP regulatory frameworks.

## 3. Applications of Exosomes

### 3.1. Exosomes in Diagnostic Applications

Increased secretion of exosomes has been reported in cancer patients; therefore, exosomal markers are emerging as attractive targets for cancer detection. Several studies have reported the increased amounts of plasma exosomes and have correlated these with tumor burden [38,39]. Several miRNAs found in exosomes derived from the blood of lung cancer patients exhibit distinct profiles compared to healthy individuals, positioning them as promising biomarkers for diagnosis, prognosis, and disease monitoring. This represents a non-invasive alternative to traditional tissue biopsies, termed a “liquid biopsy.” Exosome-derived miRNAs offer several advantages: they are stable in body fluids, disease-specific, and suitable for early-stage cancer detection [40]. In the long term, they have the potential not only to enhance diagnostic capabilities but also to enable the development of personalized treatment strategies.

Exosomal protein signatures have also been explored to differentiate lung cancer subtypes. Exosomes isolated from NSCLC patients have shown elevated levels of CD151, CD171, tetraspanin 8, alpha-2-HS-glycoprotein (AHSG), and extracellular matrix protein 1 (ECM1) [41]. In a comparative study of adenocarcinoma (AC) and squamous cell carcinoma (SCC) patients, TP63 and KRT5 were elevated in SCC, while CEACAM6 and SFTPB were elevated in AC [42]. Lipopolysaccharide-binding proteins have also been implicated as potential drivers of metastatic NSCLC [43]. Moreover, CD91 is significantly overexpressed in exosomes from NSCLC patients. These exosomes also display increased levels of SRC, EGFR, and other signal transduction proteins. Additional markers such as NY-ESO-1, EGFR, PLAP, EpCAM, and Alix have been associated with poor prognosis in NSCLC patients [41].

### 3.2. Exosomes in Prognostic Applications

Growing evidence suggests that the content and composition of patient-derived exosomes correlate strongly with lung tumor progression and metastasis. A recent study of 125 NSCLC patients (compared to four healthy individuals) in China identified serum-derived exosomal biomarkers using ultracentrifugation, followed by characterization through transmission electron microscopy (TEM), qNano analysis, and immunoblotting and showed significantly elevated levels of AHSG and ECM1 in early-stage NSCLC patients [44]. Yang et al. analyzed PD-L1 mRNA, exosomal PD-L1 (exoPD-L1), and soluble PD-L1 (sPD-L1) in NSCLC patients across malignancy stages and post-ICI treatment, compared to healthy controls. PD-L1 mRNA was elevated in males and smokers, with no link to age or metastasis. A ~1.86-fold increase in exoPD-L1 post-ICI treatment correlated with better progression-free survival, while sPD-L1 showed no such association. Combined PD-L1 mRNA and exoPD-L1 analysis may better predict treatment response and survival in NSCLC [45].

Li et al. found exoPD-L1 levels to be significantly higher in NSCLC patients than in healthy individuals, correlating with advanced stage, tumor size, lymph node involvement, and metastases. In contrast, sPD-L1 levels were linked only to tumor size, showing limited clinical relevance [46]. Pang and colleagues explored exoPD-L1 as a prognostic tool for assessing clinical response to anti-PD-1/PD-L1 therapy. While they confirmed elevated exoPD-L1 levels in NSCLC patients, no significant stage-specific differences were found. However, Li and colleagues reported that exoPD-L1 levels were higher in late-stage (Stage III/IV) compared to early-stage (Stage I/II) NSCLC patients [46,47]. This suggests that more clinical data are needed to definitively determine the predictive value of exoPD-L1 alone in lung cancer severity.

Yang and colleagues also developed an immuno-biochip capable of selectively capturing tumor-derived exosomes. This platform was used to quantify levels of miR-21 and thyroid transcription factor-1 (TTF-1) mRNA in PD-L1-positive exosomes from NSCLC patients. The method allowed clear differentiation among healthy donors, early-stage, and late-stage NSCLC patients, with a six-fold reduction in detection time compared to traditional workflows involving immunomagnetic separation, RNA isolation, and qRT-PCR [48]. Finally, Wang and colleagues identified lipopolysaccharide-binding proteins as a distinct component in circulating exosomes from NSCLC patient serum, proposing them as potential biomarkers for metastatic NSCLC [43].

### 3.3. Exosomal Proteins and miRNAs in Lung Cancer Progression

Several protein markers, including CD151, CD171, CD91, CD317, tetraspanin 8, EGFR, AHSG, and ECM1, are significantly elevated in lung cancer patients, and have been identified in exosomes [49]. Additionally, EGFR, placental alkaline phosphatase, epithelial cell adhesion molecule (EpCAM), and Alix have been reported as predictive markers for long-term overall survival [49].

Similar to normal lung epithelial cells, exosomes are also released from tumor cells via the core biogenesis processes mediated by endosomal sorting complexes required for transport (ESCRT) components, tetraspanins, and lipid-driven budding [50]. The distinguishing factor between exosomes lies in their distinct regulation of oncogenic signaling and cargo selection [51]. For example, Rab GTPase-dependent vesicle trafficking is promoted in NSCLC, leading to increased MVB–plasma membrane fusion and ultimately enhancing the exosome release in NSCLC patients [51]. Furthermore exosomes secreted by tumor cells are taken up by target cells (via direct fusion with the plasma membrane, endocytosis, micropinocytosis, phagocytosis, or specific receptor-mediated binding) where the active cargo alters gene transcription and mRNA translation thereby promoting tumor growth [52]. Thus, exosomes can serve as biomarkers due to their active cargo which differentiates them based on their cellular origin. For example, proteomic analyses of NSCLC patients with malignant pulmonary nodules revealed distinct markers (AMBP, APOA1, F5, HABP2, FGA, F9, F10, APOE, FGG, LGALS3BP, ACTG1, FGB, C4, PA, IGHG1, ITIH3, ITIH1, C4A, F2, ALB, and ITIH2). However, a major drawback of this study is that the researchers did not perform a side by side comparisons to establish baseline levels of these markers on exosomes [53].

Studies have also highlighted the diagnostic and prognostic value of selected exosomal microRNAs. For example, miR-486-5p and miR-451a have been identified as diagnostic biomarkers capable of distinguishing lung cancer patients from healthy individuals with high sensitivity and specificity [54]. Increased levels of miR-1246, miR-23b-3p, miR-10b-5p and miR-21-5p are associated with poor overall survival in NSCLC, making them reliable markers for predicting disease stage [55]. Furthermore, in another study, exosomal miR-1290 has been associated with progression-free survival; miR-382, miR-1246, miR-23b-3p, miR-21-5p, and miR-10b-5p have been linked to overall survival; and miR-21 and miR-4257 have been proposed as biomarkers for disease-free survival in lung cancer patients [54].

In addition to RNA-based markers, exosomal DNA has shown utility in identifying molecular alterations associated with lung cancer progression. These exosomal RNA and DNA signatures offer insight into histology-specific genetic mutations and differential gene expression profiles, further supporting the role of exosomes in both diagnostic and prognostic applications for lung cancer [52,56]. Researchers have identified a panel of NSCLC-specific lncRNAs within plasma exosomes, including linc01125, HNF1A-AS1, Mir100hg, linc01160, and ZNRF3-AS1 present promising biomarkers for distinguishing NSCLC patients from healthy individuals [57].

#### Exosomal Cargoes in Small-Cell Lung Cancer (SCLC)

While the majority of studies have focused on NSCLC, emerging evidence suggests that SCLC-derived exosomes possess distinct molecular signatures. SCLC is characterized by the loss of tumor suppressor genes TP53 and RB1, along with neuroendocrine differentiation markers such as chromogranin A and synaptophysin [58]. Exosomes derived from SCLC cells are enriched with specific microRNAs, including miR-375, miR-21, and miR-92a, which have been implicated in promoting tumor proliferation, angiogenesis, and immune evasion [59]. Additionally, SCLC exosomes carry neuroendocrine-associated proteins and signaling molecules that facilitate rapid metastatic dissemination and therapy resistance [60]. Compared to NSCLC, SCLC-derived exosomes exhibit enhanced capacity to modulate the tumor microenvironment and support aggressive disease progression. However, the limited availability of SCLC-specific studies highlights a critical gap that warrants further investigation to fully elucidate their diagnostic and therapeutic potential.

Collectively, these findings underscore the multifaceted role of exosomal cargoes in regulating tumor progression through diverse molecular mechanisms. Exosomal miRNAs and lncRNAs modulate gene expression by targeting key signaling pathways, including STAT3/JAK2, PI3K/AKT, and MAPK cascades, thereby promoting proliferation, invasion, and metastasis [61]. Protein cargoes such as EGFR, integrins, and tetraspanins facilitate intercellular communication and enhance tumor cell survival and migration [62]. Additionally, exosomes contribute to metabolic reprogramming by altering glycolytic pathways and promoting lactate production within the tumor microenvironment. These coordinated processes enable exosomes to act as critical mediators of tumor progression, immune modulation, and therapeutic resistance, highlighting their potential as both biomarkers and therapeutic targets.

### 3.4. Interplay Between Exosomal RNA Cargoes and Cancer Stem Cells (CSCs)

The tumor microenvironment is a complex entity that comprises cancer cells, immune cells, stromal cells and cancer stem cells (CSCs) [63]. CSCs are a subset of the cancer cell population that exhibit stemness, meaning they possess self-renewal properties and can differentiate into specialized cells [64]. These properties originate from aberrant gene expression patterns, including elevated proto-oncogene expression and reduced tumor suppressor gene expression as well as aberrant metabolic factors [65]. Often, dormant CSCs can escape encounter with effect of chemotherapy that targets proliferating cancer cells, leading to drug resistance [64]. Due to the surface biomarkers of CSCs, particularly lipid rafts and drug efflux proteins, CSCs exhibit enhanced tumor resistance [66].

Exosomes secreted by lung cancer cells containing miRNAs can activate cancer stem cells and promote tumor development or drug resistance. Reports indicate that exosomes secreted by lung cancer cells transport miR-210, which promotes phosphorylation of STAT3 and JAK2, while diminishing the expression of ten eleven translocation 2 (TET2), leading to the activation of hematopoietic cancer stem cells and enhanced tumor vasculature in a lung cancer models [67,68]. Conversely, exosomes secreted by CSCs transport certain miRNAs or long non-coding RNAs (lncRNAs) that facilitate cancer progression and metastasis in non-CSCs. The study by Shi et al. indicated that exosomal Mir100hg released from CSCs enhances the metastasis of lung cancer cells both in vitro and in vivo by upregulating ALDOA expression, increasing lactate production and H3K14 lactylation, and promoting the transcription of 169 metastasis-related genes [69]. Another study showed that exosomal lncRNA Mir100hg from cancer stem cells is transported to lung cancer cells, binding to miR-15a-5p and miR-31-5p, hence augmenting the overall glycolytic activity and metastasis of lung cancer cells [70]. Reported findings indicate that exosomes significantly facilitate communication between cancer cells and CSCs by transferring exosomal RNA, thereby promoting disease progression and serving as potential biomarkers for lung cancer prognosis.

### 3.5. Exosomes in Therapeutic Applications

**Figure 2:**
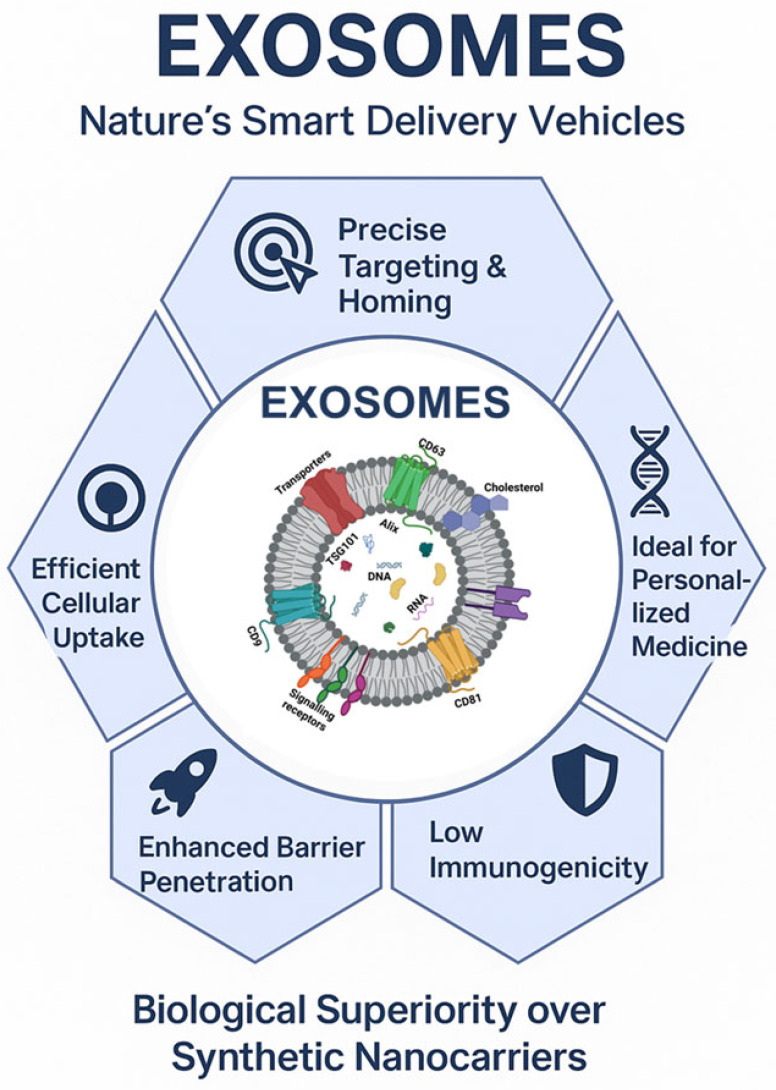
**Biological Advantages of Exosomes Over Synthetic Nanocarriers.** Schematic illustration highlighting the biological superiority of exosomes compared to synthetic nanocarriers. Exosomes possess inherent properties that make them highly effective drug delivery vehicles, including: (i) precise targeting and homing due to their surface molecules; (ii) efficient cellular uptake; (iii) enhanced barrier penetration, enabling delivery across physiological barriers; (iv) low immunogenicity, reducing adverse immune responses; and (v) suitability for personalized medicine due to their endogenous origin and customizable cargo. The exosomal membrane structure displays key surface markers and internal components (proteins, lipids, nucleic acids) contributing to their unique functional properties.

Exosomes offer vast potential in cancer treatment due to their natural ability to facilitate intercellular communication. They exhibit improved biocompatibility and reduced toxicity compared to synthetic drug delivery vehicles [71]. and hold significant potential for advancing personalized medicine. Patient-derived exosomes can be isolated, modified, and reintroduced into the patient’s system for therapeutic effects [72]. Exosome-mediated drug delivery systems offer several advantages, as depicted in Figure 2. Exosomes also exhibit low immunogenicity, reducing the risk of triggering undesirable immune responses, contributing to an improved safety profile and prolonged circulation time [73].

A major limitation of conventional therapeutics and DDS is their inability to cross biological barriers, particularly the blood–brain barrier. Exosomes, due to their nanoscale size and ability to evade lysosomal degradation via endosomal bypass, can overcome this challenge [74]. Uptake of exosomes by target cells through membrane fusion, phagocytosis, micropinocytosis, or various forms of endocytosis facilitates efficient drug delivery [75]. Naturally occurring and surface-decorated with targeting ligands, exosomes possess intrinsic trafficking capabilities and can also be engineered for enhanced tissue- or cell-specific delivery, thereby reducing systemic toxicity [74].

In cancer therapy specifically, exosomes could be utilized to target the tumor cells. They can be engineered to improve targeting, drug loading, and therapeutic efficacy and the following strategies can be used.

#### 3.5.1. Surface Modification Approaches

Surface modification techniques are crucial for enhancing the targeting ability and functionality of exosomes in therapeutic applications. Chemical modifications involve attaching functional groups or molecules to the exosomal surface using methods such as click chemistry, thiol-maleimide conjugation, EDC/NHS coupling, and amidation [76,77,78]. These strategies improve the specificity and efficiency of exosome-based delivery systems. In genetic engineering approaches, donor cells are modified to express desired surface proteins or peptides, such as human induced pluripotent stem cells (hiPSCs) engineered to express the rabies viral glycoprotein (RVG) for brain targeting [79]. However, the efficiency of genetic engineering can vary across cell types, and large-scale production remains a challenge, with the added risk of altering exosomal composition [80]. Another strategy includes fusion with targeting agents, such as peptides, antibodies, or magnetic particles. For example, neutrophil-derived exosomes functionalized with magnetic nanoparticles have shown enhanced tumor site accumulation under magnetic fields [81,82]. Dual-ligand strategies allow for simultaneous targeting of different pathways or combined therapeutic and diagnostic applications [83]. Stimuli-responsive modifications enable exosomes to respond to pH, temperature, or magnetic fields, ensuring controlled and site-specific drug release [81].

#### 3.5.2. Drug Loading Approaches

Drug loading into exosomes can be achieved through endogenous or exogenous approaches. In endogenous loading, therapeutic agents are introduced into donor cells and naturally incorporated into exosomes during their biogenesis. For example, CD8^+^ T cells engineered to express interleukin-2 (IL-2) and anti-EGFR antibodies produce exosomes with significant antitumor effects against lung cancer [84]. In exogenous loading, pre-isolated exosomes are loaded with drugs using various techniques. Incubation allows passive diffusion or surface adsorption of drugs but typically yields low efficiency [85]. Electroporation uses electrical pulses to create pores in the exosome membrane, facilitating drug entry, particularly for nucleic acids, although it may damage membrane integrity [86]. Sonication employs ultrasound waves to temporarily disrupt membranes for drug incorporation, requiring careful optimization to preserve exosome structure [87]. Extrusion involves mechanically breaking and reforming exosomes in drug-containing solutions using specialized equipment. Finally, freeze–thaw cycles repeatedly disrupt membranes to enable drug entry, although excessive cycling can compromise exosome stability [88].

## 4. Exosome-Based Therapeutics in Lung Cancer Therapy

**Figure 3:**
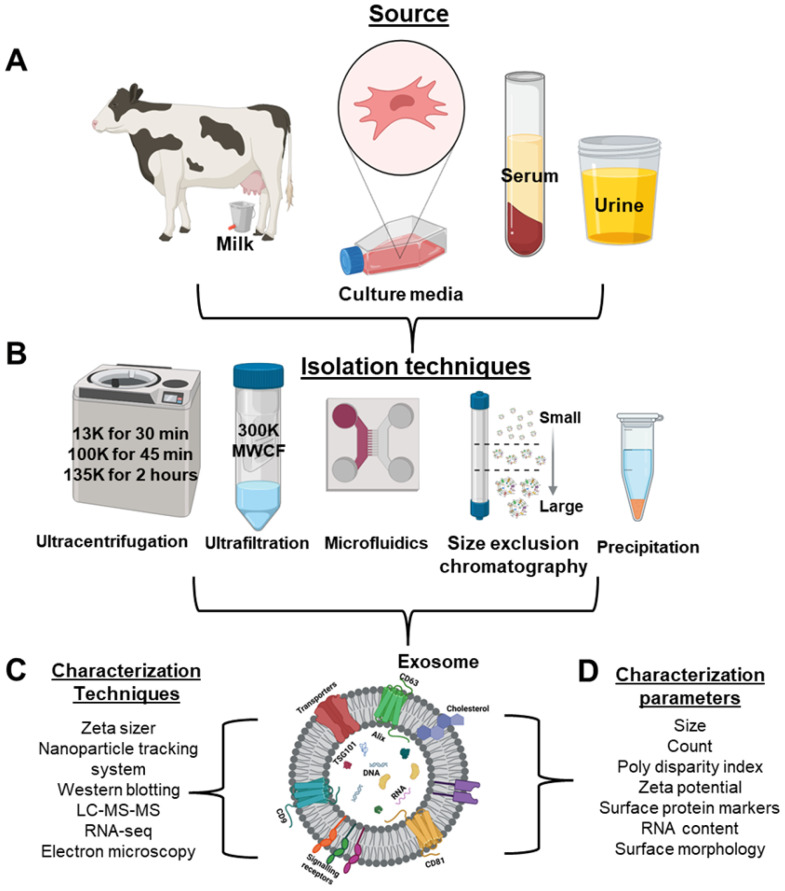
**Representative Workflow for Exosome Isolation and Characterization.** A schematic representation of the exosome research workflow is shown. (**A**) Exosomes can be derived from diverse biological sources, including cell culture media, milk, serum, and urine. (**B**) Various isolation methods such as differential ultracentrifugation, microfiltration, microfluidics, size-exclusion chromatography, and precipitation-based approaches are employed for exosome recovery. (**C**) Isolated exosomes are further characterized using multiple techniques including nanoparticle tracking analysis (NTA), dynamic light scattering (DLS), transmission electron microscopy (TEM), and Western blotting for specific exosomal markers. (**D**) Key characterization parameters include particle size, concentration, morphology, and expression of exosomal surface proteins (e.g., CD9, CD63, CD81, TSG101), ensuring accurate identification and quality assessment.

Despite numerous sources of exosomes explored in the literature, as described in Figure 3, the ideal source must be scalable, stable, biocompatible, and abundantly available. Additionally, exosomes should possess transfection potential and should not compromise the therapeutic efficacy of the loaded cargo [6]. Based on these criteria, bovine milk and colostrum-derived exosomes represent an excellent choice for drug delivery applications. Notably, bovine milk is an abundant and inexpensive source of exosomes, making it feasible for upscaling to meet clinical demands. Milk and colostrum-derived exosomes have demonstrated safety and low immunogenicity, thereby reducing the risk of adverse immune responses [85,89].

Milk exosomes are especially well suited for oral delivery systems due to their high resistance to gastrointestinal degradation and natural affinity for intestinal epithelial cells. They have shown the ability to encapsulate and deliver both hydrophilic and lipophilic small molecules as well as biologics efficiently [90]. Studies have demonstrated that drug-loaded milk exosomes improve therapeutic efficacy compared to free drugs in both in vitro and in vivo models. While most drug loading techniques are time-consuming, labor-intensive, and require sophisticated instrumentation, our laboratory has successfully achieved drug loading of ~20% through simple incubation, as confirmed by UPLC analysis. This cost-effective approach makes milk exosomes highly attractive for clinical applications.

Our laboratory demonstrated effective delivery of curcumin (CUR) using milk-derived exosomes, which exhibited antiproliferative activity in vitro, tumor inhibition in vivo [91]. Additionally, the formulation remained stable for six months at −80 °C, retaining its antiproliferative efficacy comparable to freshly prepared formulations. This exosomal approach overcame the low bioavailability of CUR, with 3–5-fold higher levels observed in the liver, lungs, and brain compared to free CUR [91]. In another study, we have demonstrated the delivery of celastrol (CEL); orally administered exosomal CEL exhibited significant tumor growth inhibition, without inducing systemic toxicity [92]. Milk exosomes have also served as carriers for berry-anthocyanidins (Anthos) [93]. Notably, ExoAnthos formulations administered intraperitoneally at half the dose of free Anthos were effective in reducing lung tumor growth, whereas the free compound remained ineffective [93]. This suggests that exosomes can enhance the therapeutic efficacy of drugs that would otherwise require higher doses to elicit a response.

Similarly, milk-derived exosomes have improved the therapeutic efficacy of standard-of-care chemotherapeutics such as paclitaxel and docetaxel [94]. Our lab has previously shown that exosomal formulations of paclitaxel not only improved therapeutic outcomes but also reduced toxicity associated with the free drug or its commercial solubilizer, Cremophor EL [85,89]. Additionally, our laboratory has demonstrated the potential of milk exosomes for the delivery of siRNA and DNA [95,96]. This system not only delivered the genetic payload effectively but also elicited a therapeutic response, while protecting nucleic acids from enzymatic degradation.

### 4.1. Approaches to Enhanced Therapeutic Efficacy—Tumor Targeting

Numerous factors influence the in vivo biodistribution and efficacy of exosomes, including their cellular origin, route of administration, and specific targeting strategies as described in Table 1. Among these, tumor targeting plays a particularly critical role and is often achieved by engineering the exosomal surface by attaching appropriate targeting ligands. Although the literature on surface modification of exosomes is still evolving, several promising approaches have been reported as described here briefly.

**Table 1:** Summary of in vivo Studies on Exosome-Based Therapeutics in Lung Cancer.

Exosomes Source	Tumor Model	Agent	Route and Frequency	Key Findings	Ref
Bovine Milk	LC A549 (*s.c.*) tumors	Withaferin A (WFA) and ExoWFA	*p.o.*, 8 mg/kg, 3 times a week	A significantly Greater tumor inhibitory with ExoWFA (74%) compared to free WFA (50%)	[94]
Bovine Milk	LC A549 (*s.c.*) tumors	Paclitaxel (PAC) and ExoPAC	PAC (*i.p*, 4 mg/kg) ExoPAC (*p.o.*, 4 mg/kg; 3 times a week)	ExoPAC (4 mg/kg) (60%) > PAC (31%)	[89]
Bovine Colostrum	LC A549 (*s.c.*) tumors	PAC, ExoPAC and FA-ExoPAC	*p.o.*, 6 mg/kg, once a week	FA-ExoPAC (54%; *p* < 0.05) > ExoPAC (45%; *p* < 0.05) > PAC (30% ns)	[85]
Bovine Colostrum	LC A549 (orthotopic) tumors	PAC, Abraxane, ExoPAC, FA-ExoPAC.	*p.o.* and *i.v.* (6 mg/kg)	i.v. FA-ExoPAC (70%; *p* < 0.001) > i.v. Abraxane (62%; *p* < 0.001) > p.o. FA-ExoPAC (39%; ns) > i.v. PAC (32%; ns)	[85]
Bovine Colostrum	LC A549 (orthotopic) tumors	PAC, Abraxane, ExoPAC, FA-ExoPAC.	*p.o.* and *i.v.* (4 mg/kg for three weeks, then switched to 8 mg/kg	i.v. FA-ExoPAC (76%; *p* < 0.001) > *p*.o. FA-ExoPAC (55%, *p* < 0.001) ≈ i.v. Abraxane (59%, *p* < 0.001) > p.o. ExoPAC (36%, *p* < 0.05) > i.v. PAC (24%) > p.o. FA-Exo (9%).	[85]
RAW 264.7 cells	LC 3LL-M27 (orthotopic) tumors	exoPTX and PTX	*i.n.* (50 mg/kg/mouse) every other day–seven treatments	A significant (*p* < 0.05) inhibition of metastases growth by exoPTX treatment was demonstrated	[87]
RAW 264.7 cells	LC 3LL-M27 (orthotopic) tumors	PTX, Exo, ExoPTX and AA-PEG-exoPTX	*i.v.* (0.5 mg/kg)	AA-PEG-vectorized exosomes loaded with PTX (AA-PEG-exoPTX) possessed a high loading capacity, profound ability to accumulate in cancer cells upon systemic administration, and survival by 25 days as compared to free PTX	[97]
Bovine milk	LC A549 (*s.c.*) tumors	Celastrol (CEL) and Exo-CEL	*p.o.* (8 mg/kg)	ExoCEL (77%; *p* = 0.001) > CEL (52%)	[92]
Bovine Colostrum	LC A549 (orthotopic) tumors	CEL, Exo-CEL, FA-Exo-CEL	*p.o.* (8 mg/kg)	FA-ExoCEL (84%; *p*< 0.001) ExoCEL (61%; *p* < 0.01) and CEL (44%; *p* < 0.05) compared to untreated	[98]
Bovine Colostrum	LC A549 (orthotopic) tumors	Cannabidiol (CBD) and FA-Exo-CBD	*p.o.* CBD (15 mg/kg); FA-Exo-CBD (7.5 mg/kg)	FA-ExoCBD (79%; *p*< 0.001) and CBD (63%; *p* < 0.01) compared to untreated	[99]
Bovine Colostrum	LC A549 (*s.c.*) tumors	EPM-siKRAS, EPM-siSCR	*i.v.* (15 μg/dose)	A significant decrease in tumor volume (67%; *p* < 0.001) and tumor weight (76%; *p* < 0.001) correlated with >85% knockdown of KRAS protein (*p* < 0.01) levels in tumors treated with FA-EPM-siKRAS	[95]
Bovine Colostrum	LC A549 (orthotopic) tumors	FA-EPM, EPM-siKRAS, and FA-EPM-siKRAS	*i.v.* (15 μg/dose) three times a week	A significant reduction in tumor growth rate by FA-EPM-siKRAS (62%, *p* < 0.001).	[95]
H1299 culture media	LC H1299 (orthotopic) tumors	PBS, Exo-SPIONs, DOX, Exo-SPIONs-DOX, Exo-SPIONs-DOX + Magnet	*i.v.* (5 mg/kg) Every two days for 18 days	Exo-SPIONs-DOX exhibited optimal tumor tissue delivery and tumor suppression in the presence of an external magnetic field and reduced the toxicity of the DOX to normal tissues.	[100]
Murine embryonic stem cells (ESCs)	LLC (orthotopic) tumors	Murine ESCs engineered to produce GM-CSF	225 µg exosomes (empty vector or GM-CSF) immunized twice (days 0 and 7), *s.c.* right flank	Vaccination reduced lung tumor burden from 1.86% in non-vaccinated, LLC-challenged mice to 0.036% in corresponding vaccinated mice.	[101]
MSC-derived exosomes fused with folate-modified liposomes	CT26 (*s.c.*) tumors	PTX	Intratumoral injections every 3 days four times	Hybrid exosomes loaded with PTX inhibited tumor growth by 60% as compared to free PTX and by 75% as compared to PBS control and significantly improved the survival benefit.	[102]
T-Cell-Derived Exosomes fused with liposomes	CT26-MSLN (orthotopic) tumors	PTX (1.5 mg/kg)	Injections every 3 days, 3 times	Growth of metastatic lung cancer was significantly inhibited by hybrid exosomes loaded with PTX	[103]
T cells expressing the chimeric antigen receptor (CAR-Exos)	MSLN-LLC (orthotopic) tumors	PTX	Aerosol inhalation for two weeks	Inhaled PTX@CAR-Exos accumulated within the tumor area, reduced tumor size, and prolonged survival with little toxicity.	[104]

*s.c.* = subcutaneous; *p.o.* = oral; *i.v.* = intravenous; *i.p* = intraperitoneal; *i.n.* = intranasal.

Folate receptors are known to be overexpressed in various lung cancer subtypes, making them an attractive target for tumor-specific delivery [95]. Our lab has previously demonstrated successful surface functionalization of milk-derived exosomes with folic acid using amine-reactive crosslinker chemistry (click chemistry), which significantly enhanced tumor targeting and antitumor efficacy [85]. Beyond this chemical approach, folate receptor-targeting chimeras (FRTACs) have also been developed to specifically bind malignant tumor cells [105].

In an interesting study, Xu and colleagues showed that folic acid-modified milk exosome-based delivery of c-Kit siRNA overcome EGFR-TKI resistance by suppressing mTOR signaling in lung cancer cells [106]. These formulations inhibited tumor growth, prolonged survival, and reversed gefitinib resistance in both xenograft and liver metastasis models. Additionally, in our studies with milk exosomes, we have shown successful loading and delivery of siRNA targeting key oncogenic pathways, including VEGF, EGFR, AKT, MAPK, and KRAS, achieving 2- to 10-fold knockdown in cancer cell models [96]. Notably, FA-functionalized, siKRAS-loaded exosomes preserved cargo integrity, showed resistance to RNase degradation and demonstrated potent lung tumor inhibition [96]. Shandilya et al. introduced an innovative natural ligand receptor-mediated loading approach using electrostatic interactions employing bovine lactoferrin conjugated with poly-L-lysine to efficiently load siRNA via interaction with negatively charged siRNA molecules [107].

Functional ligands can also be introduced into pre-isolated exosomes using the post-insertion method, offering a simpler and safer alternative to genetic engineering by avoiding potential gene-related side effects [108]. In this approach, lipid moieties can be inserted into the exosomal membrane; however, proper validation is essential to ensure successful incorporation and functional efficacy [109]. PEGylation, or modification of exosomes with polyethylene glycol (PEG), has been extensively studied, particularly in milk-derived exosomes, to enhance their stability, epithelial uptake, and mucus permeability while preserving the integrity of their therapeutic cargo [110]. PEGylation also prolongs circulation time by reducing opsonization and clearance by the mononuclear phagocyte system [110].

Exosomes can be conjugated with chemotherapeutics such as doxorubicin using pH-sensitive linkers, enabling targeted and controlled drug release specifically within acidic tumor regions [111]. Tumor environment-sensitive modifications take advantage of the acidic conditions of the tumor microenvironment, a consequence of the Warburg effect, wherein cancer cells preferentially undergo aerobic glycolysis, leading to increased lactic acid production [112]. Hyaluronic acid (HA) modification is another promising approach. Since the CD44 receptor, a HA-binding protein, is overexpressed in many cancers including lung, pancreatic, ovarian, and breast cancers, researchers have functionalized milk-derived exosomes with amphiphilic lipids and hyaluronan to enhance tumor targeting and selectivity [113,114].

Once functionalized, exosomes must effectively deliver their therapeutic payload be it small molecules, proteins, lipids, or nucleic acids to the target site. We have successfully demonstrated exosome-mediated delivery of both small and large therapeutic agents using various mouse models in vivo, achieving potent antitumor effects. These compounds include Withaferin A, Celastrol, Anthos, Docetaxel, Curcumin, and Paclitaxel each showing enhanced efficacy when delivered via exosomes compared to the free drug [85,91,92,94,115]. The exosomal formulations were found to be systemically non-toxic and may serve as adjuvant therapies alongside standard-of-care drugs [115].

### 4.2. Role of Exosomes in Chemoresistance and Therapeutic Targeting

Increasing evidence indicates that exosomes play a pivotal role in mediating chemoresistance in lung cancer [13]. Tumor-derived exosomes can transfer resistance-associated molecules, including miRNAs, lncRNAs, and drug-efflux proteins, to recipient cells, thereby enhancing survival signaling and reducing drug sensitivity [116]. For instance, exosomal miR-21 and miR-155 have been shown to activate anti-apoptotic pathways and confer resistance to chemotherapeutic agents. Additionally, exosomes can modulate the tumor microenvironment by promoting epithelial–mesenchymal transition (EMT), immune evasion, and metabolic adaptation [60].

Targeting exosome biogenesis, release, or uptake represents a promising strategy to overcome chemoresistance. Pharmacological inhibitors such as GW4869, which blocks neutral sphingomyelinase and exosome secretion, have demonstrated potential in sensitizing tumor cells to chemotherapy [117]. Similarly, inhibiting exosome uptake through receptor blockade or endocytosis inhibition may disrupt intercellular communication and reduce resistance propagation [118]. Combining exosome-targeted approaches with conventional chemotherapy or immunotherapy may therefore enhance therapeutic efficacy and improve clinical outcomes.

## 5. Role of Exosomes in Immunotherapy

Exosomes, secreted by nearly all cell types, can exert either beneficial or detrimental effects depending on their cellular origin [119]. The tumor microenvironment (TME) is a heterogeneous milieu comprising host cells, immune cells, stromal components, tumor-associated macrophages (TAMs), blood vessels, and other resident cells [120]. While tumor-derived exosomes can modulate immune responses and promote tumor proliferation [121], immune cell-derived exosomes often exert antitumor effects [122].

Exosomes released by immune cells such as B lymphocytes, dendritic cells (DCs), macrophages, natural killer (NK) cells, and T lymphocytes carry cell-specific proteins, nucleic acids, and antigens that engage with tumor cell receptors to modulate proliferation or stimulate immune responses [123,124]. Engineered exosomes carrying tumor-specific neoantigens can promote antigen-specific immunity [125]. Xu et al. demonstrated that milk-derived exosomes significantly elevated serum levels of pro-inflammatory cytokines such as TNF-α, IFN-γ, and IL-12, enhancing innate immunity and countering immunosuppression in drug-resistant tumors [106].

B cell-derived exosomes contribute to antitumor immunity via antibody-dependent cell-mediated cytotoxicity (ADCC) [126]. Heat-shocked mouse B lymphoma cell-derived exosomes (HS-Exo) carry immunologically active proteins such as HSP60, HSP90, MHC class I/II, CD40, and CD86, which activate CD8^+^ T cells and elicit robust antitumor responses [127]. Dendritic cells, as principal antigen-presenting cells, release exosomes that can activate T-cell-mediated immunity. For instance, α-fetoprotein (AFP)-expressing DC-derived exosomes induced strong antigen-specific responses and remodeled the TME in hepatocellular carcinoma models [128], while patient-specific neoantigen-loaded DC exosomes trafficked to lymph nodes and stimulated broad T- and B-cell responses for personalized immunotherapy [129].

Macrophage-derived exosomes also show therapeutic promise. M2-like TAMs, induced by IL-4, IL-10, TGF-β, and IL-13, often promote tumor progression [130,131,132,133], whereas M1 macrophages release ROS and cytokines such as TNF-α, IFN-γ, and IL-12 with antitumor effects [125]. Exosomes from M1 macrophages (M1Exo) accumulated at lymph nodes and tumor sites, activated T cells, and induced tumor regression in various models. When combined with anti-PD-1 or anti-PD-L1 therapy, M1Exo enhanced survival and reprogrammed M2 macrophages into an M1 phenotype, amplifying tumor suppression [134,135].

Natural killer (NK) cell-derived exosomes play key roles in immunosurveillance by delivering cytotoxic proteins such as granzyme A/B, perforin, FasL, and granulysin [136,137]. NK-92MI cell-derived exosomes selectively killed melanoma cells in vivo without harming normal tissue [138]. Additionally, NK exosomes enriched with miR-186 inhibited growth of MYCN-amplified neuroblastoma via TGF-β1 suppression [139] and delivered cisplatin to drug-resistant ovarian cancer cells, boosting antitumor effects and immune activation [140].

T cell-derived exosomes also contribute to immune activation. CD8^+^ cytotoxic T lymphocytes (CTLs) are major effectors of antitumor immunity, supported by CD4^+^ helper T cells [141]. Shin et al. showed that exosomes from IL-2-stimulated CD4^+^ T cells enhanced CD8^+^ T cell responses more effectively than exosomes from unstimulated cells. These exosomes were enriched in miRNAs such as miR-25-3p, miR-155-5p, miR-215-5p, and miR-375, contributing to antitumor effects in melanoma models [142]. Chimeric antigen receptor (CAR) T cell-derived exosomes carry functional CAR proteins, cytotoxic molecules, and show tumor-inhibitory activity [143]. Unlike CAR T cell therapy, which poses risks such as cytokine release syndrome, CAR T exosomes offer a safer, cell-free therapeutic option [144].

Despite promising preclinical results, challenges such as heterogeneity, lack of standardization, and delivery inefficiencies hinder the clinical translation of exosome-based immunotherapies. Continued research and innovation are crucial to fully realize their therapeutic potential in oncology.

### 5.1. Exosomes in Modulating Immune Responses in Lung Cancer

Immunotherapy has emerged as a major treatment option for lung cancer, often administered in combination with surgery, chemotherapy, and radiation [145]. However, tumor heterogeneity, immune evasion, treatment resistance, toxicity, and lack of target specificity limit the response rates and effectiveness of immunotherapeutic interventions in lung cancer [146]. To overcome these challenges, various nanomaterials, including exosomes, have been explored as delivery platforms [147].

**Figure 4:**
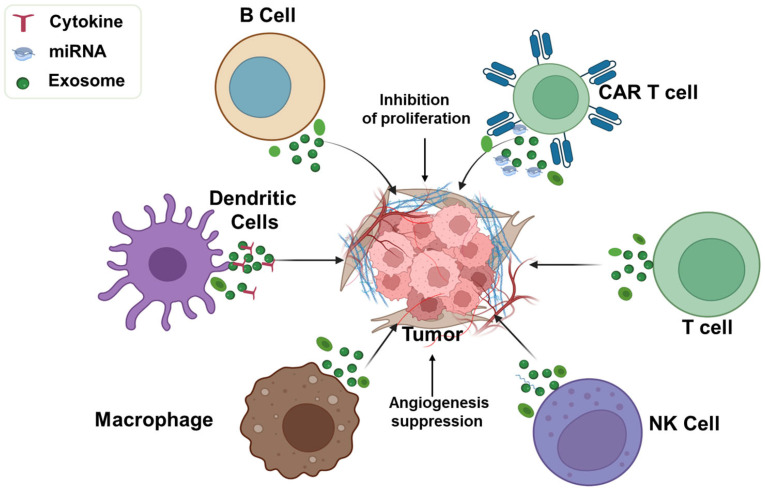
**Immune cell-derived exosomes inhibiting tumor growth.** Schematic illustration showing the role of exosomes released by various immune cells including B cells, dendritic cells, macrophages, NK cells, T cells, and CAR T cells inhibiting tumor growth. These exosomes deliver functional biomolecules such as proteins, lipids, mRNAs, and miRNAs that modulate the tumor microenvironment, enhance antitumor immune responses, and suppress tumor cell proliferation, survival, and immune evasion. The figure highlights the diverse immunomodulatory functions of exosome-mediated intercellular communication in cancer therapy.

Exosomes derived from different immune cells can inhibit tumor progression as shown in Figure 4. For instance, exosomes from human NK-92 cells activated with IL-15 and IL-21 exhibit cytotoxicity against multiple cancer cell types, including lung cancer (A549), independent of granzyme B and H expression [148]. Kang et al. demonstrated that NK cell-derived exosomes from NSCLC patients exhibited cytotoxic effects on their own circulating tumor cells (CTCs) [149]. Extracellular vesicles (EVs) from genetically engineered human CD8^+^ T cells expressing interleukin-2 and the anti-EGFR antibody cetuximab on their surfaces were shown to selectively kill lung cancer cells in an EGFR-dependent manner [84].

CAR T cell-derived exosomes, when loaded with paclitaxel and administered via inhalation, reduced lung tumor growth by increasing CD8^+^ T cell infiltration, elevating IFN-γ and TNF-α levels in the TME, and reversing immune suppression [104]. In a Phase I clinical study, exosomes isolated from dendritic cells (DEX) and loaded with MAGE tumor antigens, were administered to NSCLC patients. This resulted in enhanced MAGE-specific T cell responses, elevated NK cell cytotoxicity, and prolonged survival [150]. Another Phase I clinical trial involving DEX free of IFN-γ demonstrated NK cell activation in end-stage NSCLC patients. In a follow-up Phase II trial, second-generation DEX containing IFN-γ and MHC class I/II-restricted tumor antigens were used as maintenance immunotherapy post-chemotherapy, successfully inducing antitumor immunity via NK cells [151]. While the application of exosomes in lung cancer immunotherapy is growing, the field remains in its early stages and further research is needed to improve clinical translatability and therapeutic outcomes.

### 5.2. Potential for Delivering Immune Checkpoint Inhibitors (ICIs)

Immune checkpoints are regulatory proteins expressed on immune or tumor cells that inhibit immune responses by sending “off” signals to T cells [152]. Immune checkpoint inhibitors (ICIs), a revolutionary class of cancer immunotherapy agents, work by blocking these proteins, thereby enhancing the immune system’s ability to detect and destroy cancer cells [153]. Unlike traditional therapies, ICIs harness the body’s natural immune surveillance, as proposed by the cancer immunosurveillance hypothesis [154]. Prominent ICIs target immune checkpoints such as PD-1/PD-L1, CTLA-4, and LAG-3 [155]. ICIs often involve antibody-based therapies or RNA interference (RNAi) approaches [156,157]. For example, dendritic cells (DCs) transfected with plasmids encoding anti-PD-1 antibodies were shown to release exosomes containing membrane-bound PD-1 antibodies, which reinvigorated exhausted CD8^+^ T cells and enhanced cytotoxic T lymphocyte (CTL) responses [158]. Despite their success, ICIs are limited by off-target effects and resistance. To address this, exosomes and other nanocarriers are being investigated for the targeted delivery of ICIs directly to the TME.

Lin et al. used A549 lung cancer cell-derived exosomes to deliver PD-L1 siRNA, resulting in suppressed tumor growth [159]. Exosomes derived from HEK-293T cells were employed to co-deliver PD-L1 and CTLA-4 siRNAs, which increased CD8^+^ T cell infiltration and elevated IL-2, IFN-γ, and TNF-α levels, thereby reducing tumorigenicity in colorectal cancer models [160]. In our lab, we developed a novel EPM matrix, comprising bovine colostrum exosomes and polyethyleneimine, to deliver siPD-L1. This system successfully knocked down PD-L1 expression in A549 and LLC cells, and significantly inhibited tumor growth in an orthotopic Lewis lung carcinoma (LL/2-Luc2) mouse model [161]. While the use of exosomes for delivering ICIs remains limited, their surfaces can be modified to enhance specificity and improve delivery efficiency. Continued development in this area holds a promise for advancing cancer immunotherapy with reduced systemic toxicity and enhanced efficacy.

## 6. Exosome-Based Clinical Trials in Lung Cancer

Exosomes have emerged as highly promising candidates for treating a wide range of diseases, including cancer. Extensive preclinical studies have already demonstrated their therapeutic potential across diverse fields. In recent years, this promise has been translated into clinical investigations, with ongoing trials exploring various types of exosomes, primarily those derived from human cells. According to a survey of ClinicalTrials.gov, the primary areas of clinical focus include the use of exosomes as biomarkers, therapeutic agents, drug delivery vehicles, and components of cancer vaccines. Clinical studies summarized in Table 2 were identified through searches on PubMed, supplemented by screening of peer-reviewed clinical reports in the published literature. An advanced search was performed using combinations of the terms and the reported records were screened.

Dendritic cell (DC)-derived exosomes isolated from patient blood and loaded with MAGE peptides have been used in patients with advanced NSCLC, where each patient received a dose of 0.13 × 10^14^ exosomes containing MHC class II molecules. The peptide was loaded onto the exosomes either directly through incubation or indirectly by preloading the source DCs at a concentration of 10 μg/mL. While the exact dose of peptide administered was not reported, the treatment was well tolerated, with no evidence of severe organ toxicity or immune reactions [150].

Exosomes derived from toll-like receptor 4 ligand (TLR4L)- or interferon-gamma (IFN-γ)-matured DCs have enhanced capacity to stimulate T-cell responses compared to exosomes from immature DCs [162]. Viaud and colleagues optimized the production of DC-derived exosomes for clinical trials by maturing DCs using IFN-γ to enhance the immunogenicity of the resulting exosomes [163]. These exosomes were then loaded with MHC class I- and II-restricted cancer antigens and used as maintenance immunotherapy in a Phase II clinical study in NSCLC patients. This strategy enhanced NK cell function, and one-third of the patients experienced disease stabilization for over four months, with a median overall survival of approximately 15 months for all the patients in the study [151].

DC-derived exosomes carrying MHC–peptide complexes have shown potential in stimulating T cell responses and tumor rejection. In a Phase I clinical trial, 15 stage III/IV melanoma received autologous exosomes pulsed with MAGE3 peptides [164]. The team isolated 10^14^–10^15^ exosomal MHC class II molecules from patient blood (via leukapheresis), and each administered dose included 0.13 × 10^14^ exosomal MHC class II molecules. The exosome production process was feasible and the treatment was well tolerated with no grade II toxicity. Although MAGE3-specific T cells were not detected in peripheral blood, one patient exhibited a partial tumor response with immune activity in the tumor bed, and several others showed minor or stable responses. This study demonstrated the clinical safety and manufacturing viability of exosome-based immunotherapy [164]. Another Phase I clinical trial involved the use of mesenchymal stem cell (MSC)-derived exosomes loaded with siRNA targeting KRAS^G12D^ in patients with metastatic pancreatic cancer. This study aimed to determine the maximum tolerated dose and identify dose-limiting toxicities; however, the results have not yet been published [165].

While the therapeutic potential of exosomes is increasingly being explored, greater number of clinical trials have focused on their diagnostic applications. Exosome-based diagnostics offer significant potential for early cancer detection, monitoring disease progression, and assessing treatment response [166]. Most diagnostic studies involve isolating exosomes from patient blood samples and analyzing their molecular cargo including proteins, microRNAs, and mRNAs which reflect tumor heterogeneity and provide real-time monitoring and comprehensive tumor profiling [166].

**Table 2:** Summary of Key Clinical Studies on Exosome-Based Biomarkers in Lung Cancer Prognosis.

Clinical Application	Lung Cancer (LC)Stage	Exosome Source	Biomarkers Analyzed	Key Findings and Endpoints	Ref.
Prognostic biomarkers identification	LC stage 1–4 (*n* = 431), Control (*n* = 150)	Plasma	49 exosomal protein markers	Higher expressions of CD151, CD171, and tetraspanin 8, was the strongest differentiator between lung cancer patients and healthy participants.	[49]
Predicting anti-PD-1 immunotherapy response and prognosis	LC Stage I–III Naïve (*n* = 85), Control (*n* = 27)	Serum	Exo-PD-L1 and soluble PD-L1 (sPD-L1)	Exo-PD-L1, but not soluble PD-L1, correlated with disease progression (tumor size, lymph node status, metastasis, TNM stage).	[46]
Predicting anti-PD-1 immunotherapy response and prognosis	LC Stage I–III, patients who underwent complete anatomical resection (*n* = 363)	Serum	Tumor PD-L1 expression, serum exosomal PD-L1, and CD8+ TILs	Patients with exosomal PD-L1 ≥ 166 pg/mL tended to have worse recurrence-free survival. Serum exosomal PD-L1 levels were associated with survival in patients with NSCLC. Tumor PD-L1 expression status was significantly associated with prognosis in pathological stage I–III NSCLC.	[167]
Identifying tumor-derived exosomal biomarkers	LC (Stage not specified) (*n* = 125) Control (*n* = 46)	Serum	AHSG and ECM1 proteins	Expression levels of alpha-2-HS-glycoprotein (AHSG) and extracellular matrix protein 1 (ECM1) in serum exosomes were significantly higher in NSCLC patients than in healthy controls.	[44]
Noninvasive biomarkers for screening and prognosis of lung cancer	Adenocarcinomas (*n* = 50), Granulomas (*n* = 30) and Control (*n* = 25)	Plasma	Exosomal microRNAs	Identified four microRNAs (miR-200b-5p, miR-190b, miR-502-5p, miR-629, miR-17, and miR-100) to distinguish adenocarcinomas from granulomas.	[168]
Prognostic biomarkers in non-small-cell lung cancer	LC Stage I-IV without prior treatment (*n* = 330), Control (*n* = 312)	Serum	Exosomal miRNAs	Exosomal miR-5684 and miR-125b-5p levels are significantly down-regulated in NSCLC patients	[169]
Prognostic biomarker	newly diagnosed LC patients (*n* = 196), adenocarcinoma (*n* = 10) Control (*n* = 10)	Plasma	Exosomal miR-21, miR-17 and miR-155	Elevated levels of exosomal miR-23b-3p, miR-10b-5p and miR-21-5p were independently associated with poor overall survival	[170]
Exosomal immuno-oncological proteins as potential biomarkers to monitor response to ICIs therapy	Patients eligible for anti-PD-1/PD-L1 monotherapy or combined chemo-immunotherapy, locally advanced or metastatic LC (*n* = 17)	Serum	Tissue PD-L1, exosomal PD-L1 and exosomal PD-L2	Exosomal-PD-L1 is a more reliable diagnostic biomarker than tissue PD-L1. Exosomal-PD-L2 expression was significantly higher in tissue PD-L1-negative patients compared to tissue PD-L1-positive patients.	[171]
Rapid and reproducible exosome extraction for NSCLC Prognosis	LC adenocarcinoma (*n* = 14), interstitial pneumonia (*n* = 10) squamous cell carcinoma (*n* = 2), Control (*n* = 2)	Serum	Exosomal proteins	CD91 expression was significantly elevated on exosomes in especially lung ADC patients	[172]
Potential prognostic biomarker	Benign pulmonary disease with newly diagnosed LC NSCLC (*n* = 30) and SCLC (*n* = 8), control (*n* = 19)	Plasma	Exosomal miRNA	Exosomal miR-1290 was significantly elevated, miR-29c-3p was significantly decreased	[173]

### Challenges in Clinical Translation

Despite numerous advances and the tremendous potential of exosome-based technologies, their clinical translation continues to face significant challenges. One of the major hurdles is the lack of standardized methods, for exosome isolation and characterization. Different isolation techniques result in variability in both the efficiency and purity of exosomes, leading to inconsistencies across studies. For instance, while ultracentrifugation is suitable for processing large volumes, it is prone to co-isolating non-exosomal contaminants. In contrast, size-exclusion chromatography yields higher purity but is typically limited to small-scale batches, making it less feasible for clinical-scale applications [174].

Equally important is the characterization of exosomes, which directly affects their suitability for downstream applications. The most reliable method for exosome characterization involves detecting classical exosomal markers such as CD63, CD81, CD9, ALIX, TSG101, flotillin, and annexins through Western blot analysis [175]. Accurate characterization also requires demonstrating the absence of cell organelle-specific proteins and albumin. While Western blotting is considered the gold standard, many laboratories also use flow cytometry and ELISA-based assays, which can vary in sensitivity and specificity [176,177]. Therefore, establishing a universally accepted workflow and quality control standard for both exosome isolation and characterization is essential for ensuring consistency and reliability in clinical applications.

Another major challenge is achieving high yield and scalability in exosome production while preserving biological integrity and functional activity. Current strategies to increase exosome yield involve manipulating the biogenesis pathways through genetic engineering or optimizing culture conditions of source cells. These methods are often followed by exosome collection using bioreactor systems [178]. However, such manipulations may inadvertently alter the biological properties of exosomes or introduce risks that are not yet well understood or documented [178].

Cost is also a considerable limitation. With the current manufacturing potential, it is estimated that producing a single lot of 5 × 10^12^ human mesenchymal stem/stromal cell-derived extracellular vesicles (hMSC-EVs) for clinical trials can cost approximately $1,000,000. This quantity is sufficient for around 125 clinical dose regimens, with each regimen costing about $8000 [179]. These costs do not include the additional expenses associated with the chemotherapeutic or pharmaceutical agent being delivered, nor the cost of drug loading and formulation equipment. Collectively, these factors present significant barriers to cost-effectiveness and large-scale implementation.

In addition to technical and financial barriers, there is a lack of well-defined regulatory frameworks for exosome-based therapies. This stems from unresolved safety concerns, including risks related to contamination, immunogenicity, and batch-to-batch variability [180]. Furthermore, the field of exosome research is still in its infancy, and a lack of standardization and transparency in reporting continues to hinder progress. While developers of exosome-based mRNA delivery systems may draw inspiration from the recent regulatory success of mRNA vaccines developed by Moderna and Pfizer, it is important to note that the regulatory landscape for exosome-mediated drug delivery is likely to be more complex and nuanced [181]. Addressing these challenges will require a concerted effort from interdisciplinary experts to establish robust, transparent, and adaptable regulatory frameworks [182]. Innovations such as optimized cell culture media and advanced purification methods such as tangential flow filtration are being explored to improve scalability and quality control, but comprehensive solutions will depend on harmonizing scientific, technical, and regulatory standards across the field.

## 7. Limitations

We recognize shortcomings in the current scope of this review in establishing a balanced framework of clinical and translational applications of exosomes in lung cancer. A major limitation is that the existing literature on exosomal applications validated for lung cancer heavily relies on A549 and related NSCLC cell lines. This has resulted in overrepresentation of a single adenocarcinoma model and underrepresentation of other lung cancer subtypes, such as SCLC as well as drug-resistant scenarios. Consequently, concerns remain regarding mechanisms and generalizability of exosome applicability. Additionally, although the exosome field has advanced quite a lot in the past decade, there does not appear to be a standard protocol for exosome isolation, and characterization, especially with respect to exosome concentration (exosomal protein concentration vs particle number), which complicates cross-study comparisons and assessment of human dosages. Thus, the field needs regulatory refinement to clarify how preclinical data should be extrapolated for clinical translation. Although the literature has reported promising diagnostic and therapeutic efficacy of exosomes, clinical data remain very limited. Therefore, the direct extrapolation of these studies to humans must be interpreted with caution.

Unlike NSCLC, SCLC is often diagnosed at a later stage, when the tumor has metastasized to distant organs. Thus, it is extremely aggressive and has limited scope for surgical resection. There is a major difference in oncological mutations between NSCLC (EGFR, ALK, ROS1, BRAF, MET, KRAS) and SCLC (NOTCH, PTEN, MYC and TP53/RB1 loss) [183,184]. Along the same line, exosomes isolated from SCLC and NSCLC patients portray fundamentally distinct tumor biomarkers. While those from SCLC patients have neuroendocrine lineage and universal TP53/RB1 loss, exosomes derived from NSCLC patients exhibit diverse epithelial histologies [183,184]. This leads to distinct protein cargo and surface markers expressed on SCLC-derived exosomes (chromogranin A, synaptophysin, gastrin-releasing peptide, insulinoma-associated protein 1, thyroid-transcription factor 1, delta like canonical notch ligand 3, protein delta homologue 1, hes family BHLH transcription factor 6, MYCL proto-oncogene and CD56) [183,185]. Due to distinct parent tumors, miRNA signatures are specific to SCLC (miRNA-483-3p, miR-200b-3p, miR-3124-5p, miR-92b-5p and miR-375) and NSCLC (miRNA-152-3p, miRNA-1277-5pmiR-21-5p, miR-126-3p, miR-210-3p, miR-221-3p, Let-7b-5p, miR-146a-5p, miR-222-3p, and miR-9-5p) [186,187,188,189]. A major caveat in existing literature is that majority of the reports focus on characterizing SCLC-derived exosomes as biomarkers (metastasis, immune evasion, and macrophage M2 polarization) [186,187,188,189,190] and lack to recognize their potential in drug delivery applications.

Despite significant progress, several limitations must be acknowledged. First, a substantial proportion of preclinical studies rely on NSCLC models, particularly A549 cells, limiting the generalizability of findings across diverse lung cancer subtypes. Second, SCLC and drug-resistant models remain underrepresented, highlighting a critical gap in current research. Third, the lack of standardized protocols for exosome isolation, characterization, and quantification introduces variability across studies and hinders reproducibility. Furthermore, large-scale production and cost-effective manufacturing remain significant challenges for clinical translation. Lastly, majority of the findings are derived from preclinical studies, with limited clinical validation, emphasizing the need for well-designed clinical trials to establish safety, efficacy, and regulatory frameworks for exosome-based therapies.

## 8. Future Directions

To unlock the full potential of exosomes, it is essential to overcome several technical limitations that currently hinder their clinical application. A major undertaking is the selection of a cost-effective and scalable method for exosome isolation [181]. Ultracentrifugation remains the “gold standard” technique for exosome isolation. It offers several advantages over other techniques, including the ability to isolate exosomes based on size and density without the need for chemical additives, thereby preserving biological integrity. This method is particularly suitable for isolating exosomes from sources where large sample volumes are available including spent cell culture media, milk, colostrum and fruit juices [90]. Moreover, ultracentrifugation is highly reproducible, making it feasible for large-scale clinical applications, as reported by various laboratories [191].

Among various sources of exosomes, milk- and colostrum-derived exosomes have emerged as scalable, biocompatible, and clinically viable drug delivery vehicles, especially for lung cancer therapy [192]. Compared to other sources, bovine milk and colostrum offer natural abundance, low immunogenicity, ease of scale-up, and the ability to encapsulate a broad range of therapeutic agents [90]. Bovine milk-derived exosomes have shown enhanced delivery of chemotherapeutic agents such as paclitaxel, docetaxel, withaferin-A, celastrol, curcumin, and anthocyanidins, outperforming their free drug counterparts in efficacy [94]. Notably, milk-derived exosomes can endure gastrointestinal conditions, maintaining therapeutic efficacy when administered orally, which makes them highly suitable for non-invasive lung cancer treatments [193]. While these exosomes can potentially address systemic toxicity and poor bioavailability seen in conventional therapies, additional safety assessments in clinical trials are necessary before their widespread clinical adoption.

To improve the purity of exosomes, ultracentrifugation can be combined with newer techniques such as size-exclusion chromatography, which effectively removes protein contaminants [194]. Ongoing efforts by several research groups, including ours, aim to address batch-to-batch variability. Stability is another crucial factor for clinical logistics and storage [195]. The colloidal stability of exosomes, which contributes to their suitability for long-term storage, is governed by a high zeta potential, which minimizes aggregation [196]. Studies have shown that combining ExoQuick precipitation with ultracentrifugation can help maintain a surface potential below −25 mV, reducing aggregation and thereby enhancing shelf life [197].

In advancing exosomes as therapeutic vehicles for cancer treatment, tumor targetability is of paramount importance. Functionalizing exosomes with tumor-targeting ligands is a widely adopted strategy. For example, the overexpression of folate receptors in many tumors is exploited by functionalizing exosomes with folic acid (FA-Exo), significantly enhancing their targeting ability [95]. FA-functionalized exosomes have demonstrated superior efficiency in delivering both small molecules and nucleic acid payloads [90]. In particular, siRNA-loaded FA-Exo not only shows excellent in vitro and in vivo efficacy but also bypasses endosomal entrapment to directly release its cargo into the cytosol [198]. This targeted delivery approach has demonstrated effective gene silencing, tumor regression, and reversal of drug resistance, making it a versatile and promising strategy for future lung cancer treatment.

The tumor studies summarized in Table 1 and Table 2 represent selected preclinical and clinical studies from recent years in which exosomes from different sources were used as diagnostic and therapeutic tools for screening and mitigation of lung cancer. These reports serve as representative examples of the scope of exosomes and are not an exhaustive or universal list of all studies showing efficacy across lung cancer subtypes. While the mentioned preclinical studies highlight the potential of exosomes in inhibiting lung cancer growth, each study is restricted to the reported cell line, mouse strain, initial tumor burden and chemotherapeutic dosing regimen. Thus, further validation across different in vitro and in vivo models is required before proceeding to optimize dosage and route of administration in real-world settings.

An alternative to naturally derived exosomes is the development of artificially engineered exosomes. Although this approach is scientifically intriguing, significant challenges remain in scaling production and ensuring functional consistency. Current methods lack standardization in isolation and purification processes across different cell types [199]. Another emerging category is biomimetic exosomes, which are designed to mimic the structure and function of natural exosomes while incorporating essential bioactive components to enhance drug delivery and therapeutic outcomes [200]. However, this field is still in its early stages, and development is limited by several factors, including low production yields, absence of standardized preparation and purification protocols, challenges in achieving consistent biocompatibility and functionality, storage and cryopreservation issues, and the need for specialized equipment for large-scale production [200]. As a result, the clinical translation of biomimetic and artificial exosomes remains technically complex and under active investigation.

## 9. Conclusions

Exosomes offer significant potential as early-stage biomarkers for diagnosis and as efficient carriers for targeted drug delivery. In the context of drug delivery, exosome-based systems have shown promising results in inhibiting tumor growth and minimizing off-target toxicities, making them particularly well suited for combination therapies. In an effort to develop more patient-friendly cancer treatments, our laboratory has focused on creating tumor-targeted, exosome-based oral formulations, which have demonstrated encouraging results in in vivo studies. Based on findings from our group and others, milk and colostrum have emerged as excellent sources of exosomes due to their natural abundance, cost-effectiveness, and long-standing record of safe human consumption. Further, colostrum is enriched with bioactive molecules such as immunoglobulins and growth factors, which may enhance immune responses and improve therapeutic efficacy when used in combination with exosomal drug delivery systems though this hypothesis warrants further investigation.

Altogether, exosomes hold tremendous promise in the fields of cancer diagnostics and therapeutics and represent a compelling adjunct to conventional treatment modalities with the potential to benefit a broad patient population. Furthermore, to fully harness this potential, it is essential to establish standardized protocols for identifying and characterizing exosomal biomarkers and to integrate them into clinical practice through the development of robust and reliable databases.

## Data Availability

No new data were created or analyzed in this study. Data sharing is not applicable to this article.
